# A growth-rate composition formula for the growth of *E. coli* on co-utilized carbon substrates

**DOI:** 10.15252/msb.20145537

**Published:** 2015-04-10

**Authors:** Rutger Hermsen, Hiroyuki Okano, Conghui You, Nicole Werner, Terence Hwa

**Affiliations:** 1 Department of Physics, University of California at San DiegoLa Jolla, CA, USA; 2 TBB Group, Department of Biology, Faculty of Science, Utrecht UniversityUtrecht, the Netherlands

**Keywords:** bacterial growth, catabolite repression, metabolic coordination, mixed carbon-substrate growth

## Abstract

When bacteria are cultured in medium with multiple carbon substrates, they frequently consume these substrates simultaneously. Building on recent advances in the understanding of metabolic coordination exhibited by *Escherichia coli* cells through cAMP-Crp signaling, we show that this signaling system responds to the total carbon-uptake flux when substrates are co-utilized and derive a mathematical formula that accurately predicts the resulting growth rate, based only on the growth rates on individual substrates.

## Introduction

Bacterial cultures grown in minimal media supplemented with two carbon substrates (i.e., mixed-substrate media) can exhibit two types of behavior: In some cases, the substrates are consumed sequentially—which under the right conditions results in diauxic growth (Monod, 1942, 1947)—whereas in other cases, they are consumed simultaneously (Monod, 1942). Sequential utilization and diauxie are commonly attributed (Müller-Hill, 1996; Deutscher *et al*, 2006; Narang & Pilyugin, 2007) to catabolite repression by the cAMP-Crp regulatory system (Kolb *et al*, 1993; Busby & Ebright, 1999), even though specific studies have shown cAMP-Crp regulation to be either not necessary (Inada *et al*, 1996) or not sufficient (Okada *et al*, 1981) for diauxie. We here study the governing role of the cAMP-Crp in the simpler case of simultaneous substrate utilization.

Recently, You *et al* (2013) reported a physiological study of *E. coli* in steady-state exponential growth in minimal media supplemented with a *single* carbon substrate. To study the role of cAMP-Crp, which activates a large number of carbon-catabolic genes, the expression of the well-studied *lac* system (with LacI inactivated by IPTG) was used as a reporter of cAMP-Crp activity. It was found that LacZ expression level (*E*_z_) exhibits a negative linear correlation with the growth rate (*λ*) when the carbon substrate in the medium is varied: 

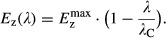
1

**Figure 1:**
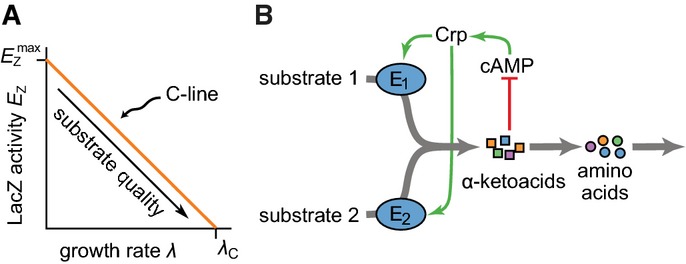
C-line and negative feedback regulation of carbon uptake

Here, *λ*_C_ is the horizontal intercept, as illustrated in Fig1A. Several other Crp-activated catabolic operons were studied; they each show a similar linear relation, each with a horizontal intercept *λ*_C_ of 1.1 to 1.2/h. These results suggest that this ‘C-line’ is a common response pattern exhibited by carbon-catabolic genes under variation of the carbon influx, mediated by cAMP-Crp regulation. Indeed, the cAMP excretion rate, a proxy for the intracellular cAMP level, shows the same trend and intercept, and removal of Crp binding obliterates the C-line.

**Figure 2:**
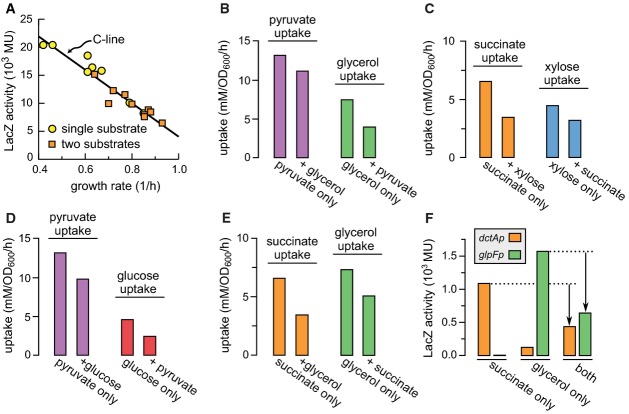
Testing model assumptions
A LacZ expression level (in activity per OD_600_ or Miller Unit) of *E. coli* K-12 cells grown in minimal medium with various carbon substrates and IPTG is plotted against the growth rate of the culture. Yellow circles and orange squares respectively represent results for growth on one (shaded entries in Table1) or two carbon substrates (clear entries in Table1). Both sets of results align along a single line (linear regression: *R*^2^ = 0.92, *P* < 10^−7^ called the C-line, described by equation 1 with horizontal intercept *λ*_C_ = 1.16 ± 0.05/h (see Fig1A).
B The uptake rates of pyruvate and glycerol, as measured during growth on pyruvate only, glycerol only, or both. As expected from the negative feedback loop illustrated in Supplementary Fig S1, the uptake of either substrate is reduced in the presence of the other. (Reported are averages over two experiments, which never deviated more than 5% from the mean.)

Illustration of the ‘C-line’ expressed in equation 1 and verified in Fig2A. In the presence of IPTG, the expression of LacZ is a reporter for cAMP-Crp activation. Under variation of the carbon substrate provided in the medium, it correlates negatively with the growth rate. Other Crp-regulated carbon-catabolic enzymes show a similar behavior, with similar horizontal intercept *λ*_C_ (You *et al*, 2013; Hui *et al*, 2015).

Regulatory mechanism responsible for the C-line. In a coarse-grained view of metabolism, carbon substrates are converted to precursors that are subsequently used in anabolic processes. Amino-acid synthesis uses a special class of precursors, the α-ketoacids. Several prominent members of these inhibit the synthesis of cAMP, thereby reducing the activity of cAMP-Crp (You *et al*, 2013). This results in a non-specific negative feedback regulation of each uptake system by the total carbon flux via cAMP-Crp: If, given the current growth rate, the *total* carbon influx exceeds the demand for α-ketoacids, carbon-catabolic gene expression is uniformly reduced.

C–E As (B), but for succinate + xylose (C), pyruvate + glucose (D), and succinate + glycerol (E). See Supplementary Fig S3 for more examples.

F Expression of *lacZ* reporter genes driven by the *dctAp* and *glpFp* promoters, which respectively control the uptake systems of succinate and glycerol. In the presence of glycerol, the expression of the succinate uptake system is markedly reduced and vice versa (black arrows).

Source data are available online for this figure.

Functionally, the C-line reflects the cell's coordination of its proteome in response to the different demands for ribosomes and metabolic enzymes at different growth rates (Scott *et al*, 2010; You *et al*, 2013; Chubukov *et al*, 2014). During fast growth, a large fraction of the cell's proteome must be allocated toward ribosomal proteins and anabolic enzymes; therefore, a reduced expression of carbon-catabolic enzymes is obligatory. Mechanistically, this reduction results from an inhibitory effect of several α-ketoacids on the synthesis of cAMP by adenylate cyclase (see Fig1B) (You *et al*, 2013). Here, we apply these insights to growth on two carbon substrates to derive a formula that predicts the resulting growth rate.

## Results and Discussion

The proposed theory of mixed-substrate usage is based on three ingredients

First, let the expression level of the catabolic enzymes for the two carbon substrates, C_1_ and C_2_, be *E*_1_ and *E*_2_. Then, at saturating substrate concentrations, the carbon uptake flux *J*_*i*_ for substrate C_*i*_ is given by 

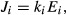
2where *k*_*i*_ is the kinetic constant.

Second, a larger growth rate requires a larger carbon uptake flux. We expect that both substrates contribute to the production of biomass, such that the resulting growth rate *λ*_12_ obeys 

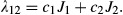
3

Here, the constants *c*_*i*_ reflect the carbon efficiency of growth on substrate C_*i*_, that is, the amount of that substrate that must be consumed to support a given growth rate. In writing equation 3, we allow these efficiencies to differ between substrates, but assume that they do not change drastically as a function of the growth rate (over the range of growth rates studied). At very high growth rates, this is likely inaccurate due to increased carbon excretion in the form of, for example, acetate (el-Mansi & Holms, 1989; Han *et al*, 1992); however, because the rate of acetate excretion is generally small compared to the rate of carbon uptake (Han *et al*, 1992), equation 3 should remain a reasonable approximation.

Third, since the driver of cAMP-Crp regulation is established to be the α-ketoacids (You *et al*, 2013), which we hypothesize respond to the *total* carbon influx regardless of whether it originates from a single or multiple substrates (Fig1B), we expect the C-line observed for single-substrate growth to hold also during growth on *two* substrates (as validated below). More precisely, we expect that *E*_1_(*λ*) ∞ *E*_2_(*λ*) ∞ *E*_z_(*λ*), so that 

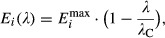
4for each substrate C_*i*_. The horizontal intercept *λ*_C_ is expected to be the same for all substrates, but the vertical intercept 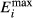 is substrate specific. Equation 4 describes a cAMP-Crp response that is a function of the growth rate only, regardless of the number of substrates. It assumes that the expression levels are not affected by any regulators other than cAMP-Crp.

The system described by equations (2,3,4) is illustrated in Supplementary Fig S1. Each substrate contributes to the total carbon-uptake flux (gray arrow). The carbon-uptake flux *J*_*i*_ of substrate C_*i*_ is proportional to the expression of the responsible enzymes *E*_*i*_ (equation 2). If the total carbon flux is increased, for example, by adding a co-utilizable substrate to the growth medium, this permits a higher growth rate *λ* (equation 3). However, an increased growth rate entails a reduced expression of the catabolic enzymes (red inhibitory lines), prompted by the increased α-ketoacid pools (Fig 1B) and quantified by the C-line (equation 4); this reduces both fluxes *J*_*i*_. Combined, equations (2–4) describe a cAMP-Crp-mediated negative feedback loop; they exploit the C-line to quantitatively describe the feedback regulation shown in Fig1B.

To derive an expression for the resulting growth rate, we first manipulate equations (3,4) to obtain 

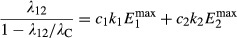
5For growth on a single substrate *C*_*i*_ (with growth rate *λ*_i_ = *c*_*i*_ *J*_*i*_), the same equations yield 

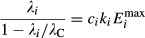
6Substituting equation 6 into equation 5, we obtain: 

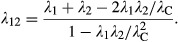
7This growth-rate composition formula is the primary result of this report. It provides a quantitative prediction of the growth rate on two co-utilized carbon substrates, based on the growth rates on each substrate alone. Remarkably, it depends on just a single (strain-dependent) parameter, *λ*_C_, whose value was already estimated above based on data from single-substrate growth. The formula therefore has no tunable parameters whatsoever.

We stress that the above model includes cAMP-Crp signaling as the only mechanism regulating catabolic enzymes; equation 7 can therefore be interpreted as a null expectation, obeyed in the absence of additional layers of regulation. That said, for many pairs of substrates, no additional regulation is known.

To validate this theory of mixed-substrate growth experimentally, we repeated the physiological study of catabolite repression in *E. coli* K12 cells as done before (You *et al*, 2013), but now for growth on 23 *pairs* of substrates, listed in Table1. We selected these as follows. We previously noticed that substrates that merge into the upper part of glycolysis (‘upper’ substrates) are often co-utilized with those entering at the bottom or directly into the TCA cycle (‘lower’ substrates) (You *et al*, 2013). Therefore, we combined three ‘lower’ substrates (succinate, pyruvate, and oxaloacetate) with five ‘upper’ substrates (mannose, xylose, glycerol, maltose, and glucose) to form 15 combinations, referred to as group A. In all these cases, the growth rate on two substrates was larger than on either substrate alone, strongly suggesting some degree of co-utilization (see Table1). For six of these 15 substrate pairs (green entries to the left of the vertical line in Table1), we also verified their co-utilization by measuring the uptake of both substrates from the growth medium (see Supplementary Fig S2, first two columns). For comparison, we also paired the five ‘upper’ substrates mentioned above with either glucose or glycerol (group B). Glucose uptake is known to inhibit the uptake of many ‘upper’ substrates (verified in Supplementary Fig S2, 4^th^ column) through the ‘inducer exclusion’ effect mediated by PTS enzyme EIIA^glc^ (Postma *et al*, 1984). Glycerol uptake is suppressed in the presence of other ‘upper’ substrates through feedback inhibition mediated by fructose-1,6-biphosphate, a key glycolytic intermediate (Zwaig & Lin, 1966). As a result of these additional interactions, the pairs in group B are *not* expected to obey the above theory, while those in group A *may,* provided the relevant uptake systems are not subject to unknown regulation in addition to cAMP-Crp.

**Table 1:** Steady-state exponential growth rates for *E. coli* K-12 strain (NCM3722).

Growth rate (1/h)		Group A			Group B	
		Succinate	Pyruvate	Oxaloacetate	Glycerol	Glucose
	alone	0.46	0.61	0.79	0.63	0.85
Mannose	0.42	0.64	0.70	0.87	0.65	0.84
Xylose	0.61	0.71	0.80	0.88	0.64	0.84
Glycerol	0.63	0.73	0.85	0.93	–	0.84
Maltose	0.67	0.77	0.85	0.90	0.70	0.84
Glucose	0.85	0.86	0.88	0.94	0.84	–

Growth rates in minimal medium with one or two carbon substrates (shaded and clear entries, respectively). All numbers are averages over two to four experiments; variability between independent experiments is of the order of 5%. Substrate pairs in group A combine one ‘upper’ substrate (i.e., a substrate merging into the upper part of glycolysis) with one ‘lower’ substrate (succinate, pyruvate, oxaloacetate). These substrate pairs are likely co-utilized: In all cases, the growth rate on both substrates is larger than on either substrate alone; for a number of cases in this group (green entries), co-utilization is directly confirmed by measuring the uptake of each substrate (see Supplementary Fig S2). For comparison, we include a second group of substrate combinations (group B), in which glycerol or glucose is paired with other ‘upper’ substrates. For none of the entries in this group does the growth rate on two substrates substantially exceed the larger of the growth rates on single substrate. This is expected from known interactions: Glucose uptake is known to inhibit the uptake of other ‘upper’ substrates through the inducer exclusion effect (Postma *et al*, 1984), as we verified by measuring the uptake of both substrates (red entries; see Supplementary Fig S2). Glycerol uptake is limited in the presence of other ‘upper’ substrates through feedback inhibition by glycolytic intermediate fructose-1,6-biphosphate (Zwaig & Lin, 1966); this leads to limited co-utilization (green entries in the column ‘glycerol’) or sequential utilization (red entry) depending on the second substrate (see Supplementary Fig S2).

We first tested the hypothesis that catabolic gene expression remains on the C-line during growth on multiple substrates (equation 4); this is expected for both groups A and B if the cAMP-Crp system indeed responds only to the total carbon flux. Fig2A confirms this: The expression of LacZ during mixed-substrate (orange squares) and single-substrate (yellow circles) growth is well described by a single C-line. This result is the first direct confirmation of an important regulatory strategy implied by previous findings (You *et al*, 2013): that cAMP-Crp responds to the total carbon-uptake flux of the cell rather than to the availability of particular carbon substrates, as commonly thought.

An important consequence of the global negative feedback illustrated in Fig1B and Supplementary Fig S1 is that the uptake of one carbon substrate should indirectly reduce the uptake of a second. We tested this for six substrate pairs from group A. In all cases, the measured uptake of each substrate was reduced in the presence of the other (see Fig2B–E and Supplementary Fig S3A and B). In contrast, this was not the case for examples from group B (Supplementary Fig S3C–E).

In our model, the reduced uptake in the presence of another co-utilized substrate follows from a reduced expression of the substrate's uptake system. To test this, we constructed two strains (strains NQ360 and NQ1513; see Supplementary Table S1) that report the expression of the *glpFp* and *dctAp* promoters, respectively driving transcription of genes encoding the glycerol and succinate uptake systems (Fig2F). During growth on both glycerol and succinate, reporter expression from each promoter was much lower than during growth on the corresponding substrates alone (black arrows).

**Figure 3:**
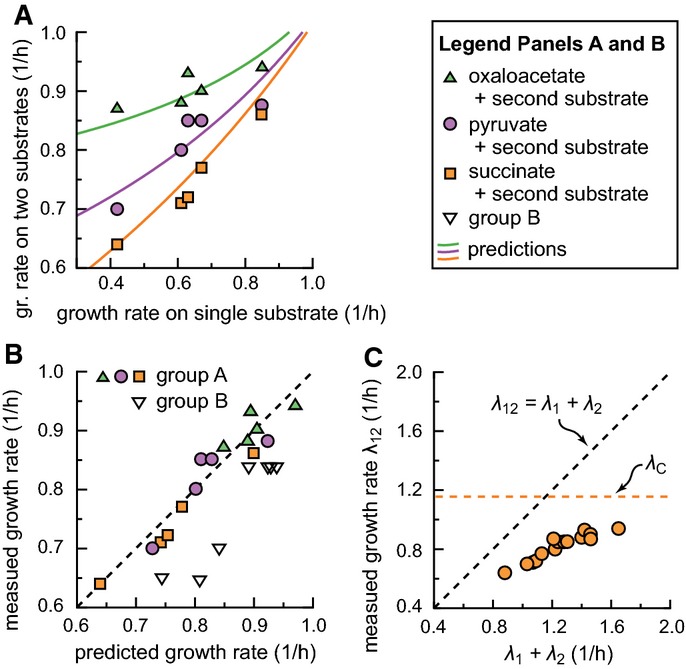
Testing the growth-rate predictions

Next, we compare the predicted growth rates on mixed substrates to the measured values in Table1. In Fig3A, the growth rates on mixed substrates are plotted against the growth rate on the ‘upper’ substrate alone, with the ‘lower’ substrate fixed to be succinate (orange squares), pyruvate (purple circles), or oxaloacetate (green triangles). Solid lines indicate the prediction based on equation 7. There is excellent agreement between theory and experiment. This is also seen in Fig3B, where all measured growth rates are plotted against their predicted values, for both group A (filled symbols) and group B (open symbols). The data for group A align with the diagonal (their regression line has slope 0.99 ± 0.18 and offset (0.00 ± 0.07)/h (95% CI), with *R*^2^ = 0.92), indicating that the theory accurately predicts the measured values. All outliers belong to group B.

The measured growth rates for cultures grown on two co-utilized carbon substrates (Table1) are plotted against the growth rates on one of the substrates, with the other substrate being succinate (orange squares), pyruvate (purple circles), or oxaloacetate (green triangles). Solid lines indicate the predictions based on the growth-rate composition formula (equation 7) and the measured growth rates on single substrates. Plotted values are averages between 2 and 4 experiments; variation between independent experiments was of the order of 5%.

The measured growth rates for cultures grown on two substrates are plotted against the corresponding theoretical predictions. Filled symbols are for group A and open symbols for group B (see Table1). Good agreement between measurement and prediction is apparent, as all filled symbols are found along the diagonal line (the linear regression line has slope 0.99 ± 0.18 and offset 0.00 ± 0.07 (95% CI), with *R*^2^ = 0.92; the distribution of the residuals is consistent with a normal distribution). The relative deviations for group B are larger than for group A: respectively 12% and 3% on average (Mann–Whitney *U*-test: *P < *2 × 10^−5^).

The growth rate *λ*_12_ on two carbon substrates is always smaller than *λ*_1_ + *λ*_2_, the sum of the growth rates on each substrate alone. The maximum growth rate possible according to the theory, *λ*_C_, is shown as the orange dashed line.

Source data are available online for this figure.

An important feature of equation 7 is that the growth rate on mixed substrates *λ*_12_ is always smaller than a direct sum of *λ*_1_ and *λ*_2_ (see Fig3C). Also, even if the single-substrate growth rates are already close to *λ*_C_, the mixed-substrate growth rate never exceeds *λ*_C_ (horizontal dotted line in Fig3C), illustrating that *λ*_C_ acts as a ‘speed limit’ for carbon-limited growth. This limit reflects that, even in the hypothetical situation in which the cell does not need to express *any* catabolic enzymes to achieve the required carbon uptake flux (*E*_*i*_ = 0), the growth rate would still be finite, limited by the finite rate of protein synthesis, anabolic processes, and the uptake of other elements.

In biotechnological applications, mixed-substrate growth is often preferred (Harder & Dijkhuizen, 1982; Joshua *et al*, 2011; Nakashima & Tamura, 2012). The growth-rate composition formula derived here, taking into account only the effect exerted by cAMP-Crp regulation on the expression of all catabolic systems (You *et al*, 2013; Hui *et al*, 2015), serves as the baseline for this important mode of bacterial growth. Deviations from this baseline suggest the presence of additional interactions affecting growth, such as inducer exclusion (Deutscher *et al*, 2006) or metabolic feedback inhibition (Zwaig & Lin, 1966; Zwaig *et al*, 1970) (see also the discussion in the caption of Table1). Cases where additional interactions occur could therefore be found by identifying substrate pairs that do not obey the theory. Such interactions may be described as modulations on top of the null-model presented here. This approach could also provide a new understanding of the classical phenomena of diauxie and sequential utilization.

## Materials and Methods

### Strain information

The strains used in the physiological study are described in Supplementary Table S1. NQ360 was made by moving the *glpFp-lacZ* construct (You *et al*, 2013) into NCM3722 by P1 transduction.

### Construction of chromosomal *dctAp*-*lacZ*

The *dctA* promoter (*dctAp*) region containing the first 20-amino-acid sequence (−283 to + 111 relative to the transcriptional start site) was PCR amplified from *E. coli* MG1655 chromosomal DNA using the PdctA-Xho-F and PdctA-ER-R oligonucleotides (Supplementary Table S2). The PCR product was cloned as an XhoI-EcoRI fragment on pKD13-*rrnBt*:P_Ltet-O1_ (Klumpp *et al*, 2009), yielding pKDT-*dctAp*. A tandem array of *kan* gene, *rrnB* terminator, and *dctAp* (*kan*:*rrnB*t:*dctAp*) on this plasmid was amplified by primers PdctA-Z-P1 and PdctA-Z-P2 (Supplementary Table S2). PdctA-Z-P1 contains a 50-bp region that is homologous to the *lacI* promoter region, while PdctA-Z-P2 contains a 51-bp region that is homologous to the first 50-bp region of the *lacZ* structural gene. The PCR product was used to transform the strain NQ309 (You *et al*, 2013) by using the *λ* Red system (Datsenko & Wanner, 2000). Substitution of *kan*:*rrnB*t:*dctAp* for the *lacI* and *lacZ* promoters was verified by PCR and subsequent DNA sequencing. NQ1513 was made by moving the construct *kan*:*rrnB*t:*dctAp:lacZ* into NCM3722 by P1 transduction.

### Growth conditions and β-galactosidase activity assays

Batch culture growth and β-galactosidase activity assays were performed as described previously (You *et al*, 2013). The culture medium used was N^−^C^−^ minimal medium (Csonka *et al*, 1994) supplemented with 20 mM NH_4_Cl, saturating amounts of either a single carbon substrate or a combination of two carbon substrates, and 1 mM isopropyl β-D-1-thiogalactopyranoside (IPTG). The concentrations of the various carbon substrates used were as follows: 20 mM fumarate, 20 mM mannose, 15 mM succinate, 20 mM fructose, 20 mM xylose, 20 mM pyruvate, 0.4% (v/v) glycerol, 20 mM maltose, 20 mM oxaloacetate, 0.4% (w/v) glucose, and 0.2% (w/v) lactose.

### Carbon-substrate uptake assays

Carbon substrate concentrations in the medium were measured as follows. The cells were grown on N^−^C^−^ minimal medium supplemented with 20 mM NH_4_Cl, 1 mM IPTG, and one or two carbon substrates. The concentrations of carbon substrates used were as follows: 6 mM glucose, 8 mM glycerol, 8 mM xylose, 3 mM maltose, 6 mM mannose, 15 mM succinate, and 20 mM pyruvate. (The growth rates at these concentrations are almost identical with those at the concentrations used for Table1.) A fraction of the exponentially growing culture was subjected to centrifugation at 16,110 *g* for 1 min; the supernatant was stored frozen at −80°C. Commercially available kits were used to measure glucose (GAHK20; Sigma-Aldrich; St Louis, MO, USA), glycerol (FG0100; Sigma-Aldrich), xylose (K-XYLOSE; Megazyme), mannose (K-MANGL; Megazyme; Bray, Ireland), succinate (K-SUCC; Megazyme; Bray, Ireland), and pyruvate (K-PYRUV; Megazyme; Bray, Ireland) according to manufacturer's instruction. For the maltose assay, maltose was first digested by incubating 5 μl samples with 30 μl of 100 mM potassium phosphate buffer, pH 6.5, containing 16.7 U/ml α-glucosidase (G0660; Sigma-Aldrich) and 38.5 mM β-mercaptoethanol at 37°C for 30 min. The released glucose was measured using the glucose assay described above. The carbon uptake rate was calculated as the slope of the plot of carbon substrate concentrations versus OD_600_ multiplied by the specific growth rate.

## Supporting Information

Source Data for Supplementary Figure S3

Review Process File
