# Supplementary figures and images for: A growth-rate composition formula for the growth of E. coli on co-utilized carbon substrates
Source: Mol Syst Biol. 2015 Apr 10;11(4):801. doi: 10.15252/msb.20145537 (PMC4422558; doi:10.15252/msb.20145537)

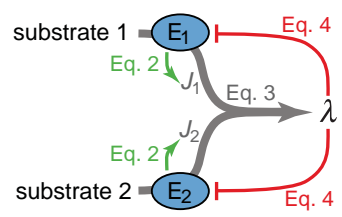

Supplement: Supplementary file 1 [file msb0011-0801-sd1.pdf]

group A

group B

succinate

pyruvate

glycerol

glucose

mannose

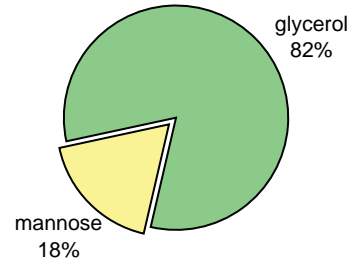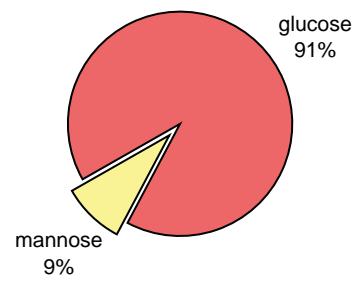

xylose

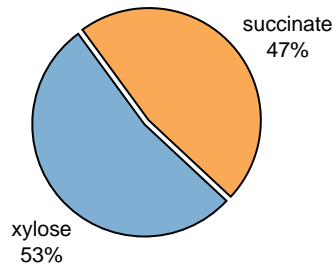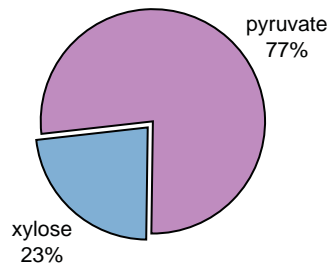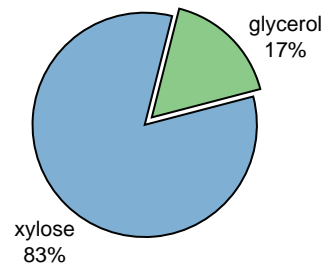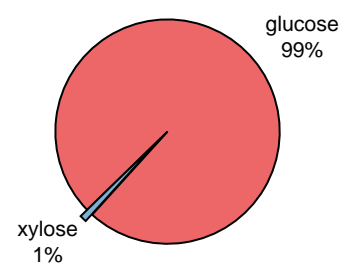

glycerol

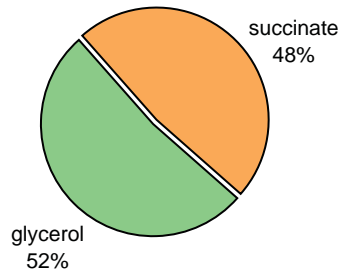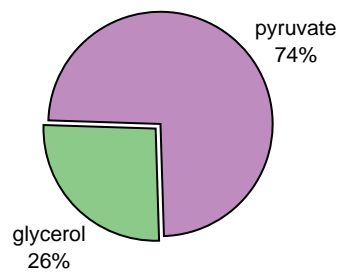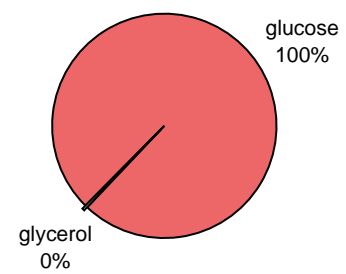

maltose

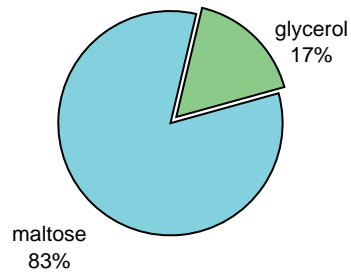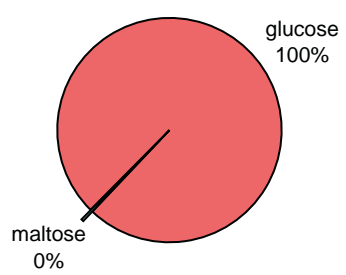

glucose

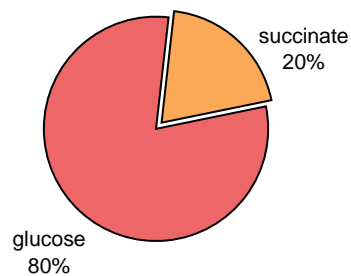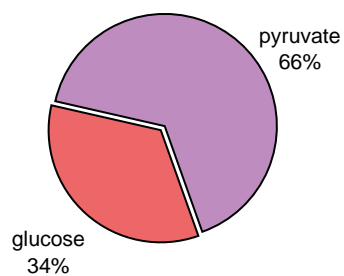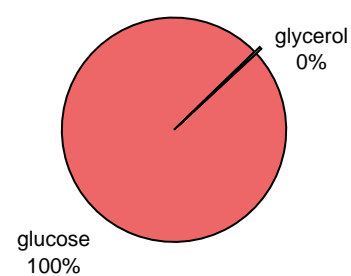

Supplement: Supplementary file 2 [file msb0011-0801-sd2.pdf]

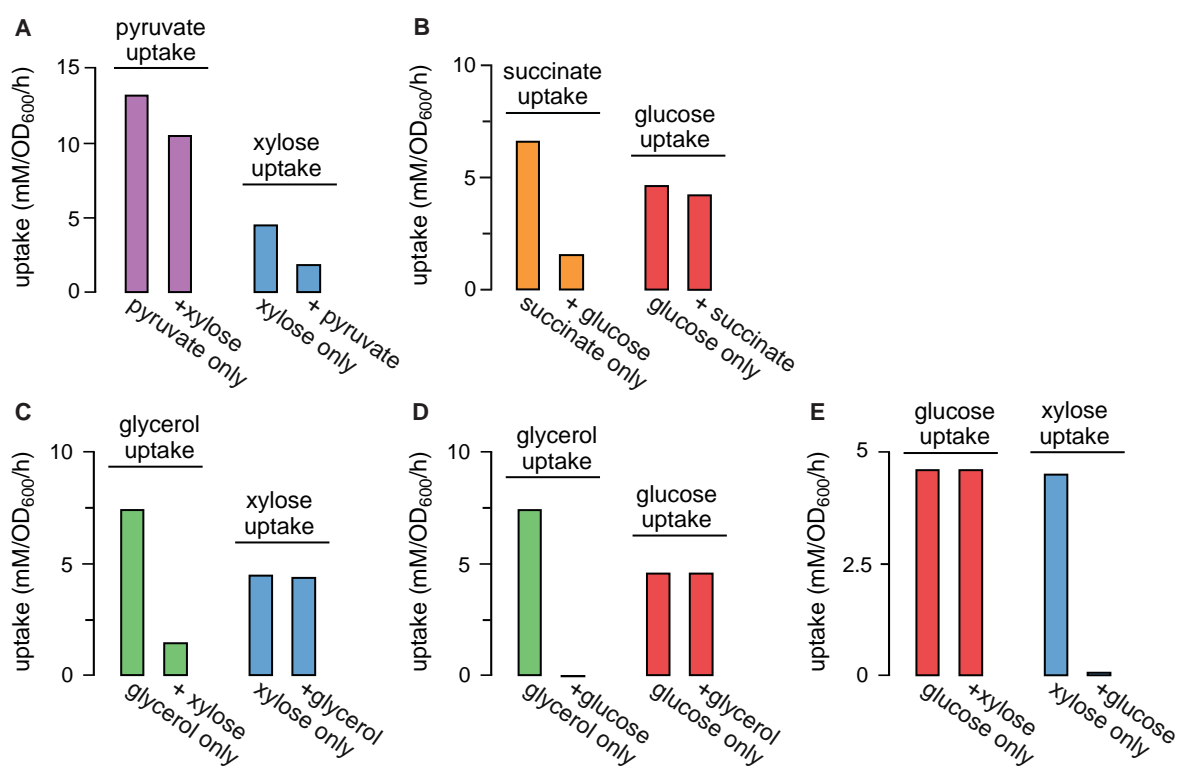

Supplement: Supplementary file 3 [file msb0011-0801-sd3.pdf]
