# Supplementary material for: A growth-rate composition formula for the growth of E. coli on co-utilized carbon substrates
Source: Mol Syst Biol. 2015 Apr 10;11(4):801. doi: 10.15252/msb.20145537 (PMC4422558; doi:10.15252/msb.20145537)
Supplement: Supplementary file 4 [file msb0011-0801-sd4.docx]

**Supplementary Figure legends**

**Figure S1. Illustration of the proposed theory of mixed-substrate growth.**

The proposed theory is fully defined by Eqs. 2 to 4. The two substrates contribute a carbon uptake flux $J_{1}$ and $J_{2}$ to the total carbon uptake (gray arrows). A larger expression of the carbon-catabolic enzymes $E_{i}$ results in a larger uptake flux (Eq. 2). We assume that a larger carbon influx can support a higher growth rate (Eq. 3). At a higher growth rate, however, the expression of carbon-catabolic enzymes is reduced, in accordance with the C-line (Eq. 4). Together, these relations form a negative feedback loop for carbon uptake, mediated by cAMP-Crp signaling.

**Figure S2. Substrate-uptake rates during growth on two substrates.**

For 13 substrate pairs, we measured the uptake rate of each substrate during steady-state balanced growth. The figure shows a pie chart for each of these pairs, visualizing which fraction of the carbon atoms consumed was derived from either substrate. When “upper” substrates (*i.e.*, substrates merging into upper glycolysis, here represented by mannose, xylose, glycerol, maltose, glucose) are combined with “lower” substrates (succinate or pyruvate; first two columns), both substrates contribute substantially to the total carbon uptake; that is, the substrates are co-utilized. In contrast, glucose suppresses the uptake of other “upper” substrates, resulting in negligible co-utilization (last column). In the case of glycerol, feedback inhibition by glycolytic intermediate fructose-1,6-biphosphate (FBP) reduces glycerol uptake in the presence of other “upper” substrates, which can result in limited co-utilization or sequential utilization depending on the second substrate (Zwaig & Lin, 1966).

**Figure S3. Reduced substrate uptake in the presence of a second substrate.**

The proposed theory of simultaneous substrate utilization predicts that, due to the global negative feedback loop illustrated in Supplementary Fig. S1, the uptake of each substrate should be reduced in the presence of a second (co-utilized) substrate. Figs. 2B-E show four examples of this phenomenon; two more examples are shown here, in Panels A and B. (In Panel B, the reduction in glucose uptake in the presence of succinate is small; this is expected given the low growth rate on succinate.) Panels C-E present contrasting results for substrate pairs of group B, which are not expected to obey the proposed theory. Panels C and D demonstrate that, while glycerol uptake is much reduced in the presence of xylose or glucose, the converse is not true: xylose and glucose uptake rates are unaffected by the presence of glycerol. (Glycerol and xylose support similar growth rates, and therefore the proposed theory would predict that they affect each other symmetrically.) Similarly, Panel E shows that xylose uptake is completely inhibited by glucose, but glucose uptake is not affected by the presence of xylose.
