# Supplementary material for: A growth-rate composition formula for the growth of E. coli on co-utilized carbon substrates
Source: Mol Syst Biol. 2015 Apr 10;11(4):801. doi: 10.15252/msb.20145537 (PMC4422558; doi:10.15252/msb.20145537)
Supplement: Supplementary file 5 [file msb0011-0801-sd5.docx]

**Supplementary Tables**

| **Strain** | **Genotype** | **Description** |
| --- | --- | --- |
| NCM3722 | wild-type *E. coli* K12 strain | The parental wild-type strain |
| NQ360 | Φ(*lacI*:*lacZp*):: Φ(*kan*:*rrnBt*:*glpFp*) placY | The *lacZ* reporter strain of *glpF* promoter |
| NQ1513 | Φ (*lacI*:*lacZp*):: Φ(*kan*:*rrnBt*:*dctAp*) tAp3t | The *lacZ* reporter strain of *dctA* promoter |
| NQ309 | Δ*lacI* Δ*lacY*  Δ*galK* Δ *ryhB* | You et al, 2013 |

**Table S1. List of strains used in the physiological study**

| **Name** | **Sequence** |
| --- | --- |
| PdctA-Xho-F | TATCTCGAGCCTGAACTTACAAATGATTGCCGAAG |
| PdctA-ER-R | AATGAATTCAGGATAGAAATGGCCAAGGAGAATAC |
| PdctA-Z-P1 | GCATTTACGTTGACACCATCGAATGGCGCAAAACCTTTCGCGGTATGTGTAGGCTGGAGCTGCTTC |
| PdctA-Z-P2 | CCAGTCACGACGTTGTAAAACGACGGCCAGTGAATCCGTAATCATGGTCATACCAATGGCTATCGCTGTCAGGACCTG |

**Table S2. List of oligonucleotides.**
